# Pain, Function, and Satisfaction After Total Knee Arthroplasty, with or Without Bariatric Surgery

**DOI:** 10.1007/s11695-022-05912-5

**Published:** 2022-01-27

**Authors:** Perna Ighani Arani, Per Wretenberg, Johan Ottosson, Annette W-Dahl

**Affiliations:** 1 Department of Orthopedics, Örebro University Hospital, Örebro, Sweden; 2 Faculty of Medicine and Health, School of Medical Sciences, Örebro University, 702 81 Örebro, Sweden; 3 Department of Surgery, Örebro University Hospital, Örebro, Sweden; 4 Scandinavian Obesity Surgery Registry, Örebro, Sweden; 5 Department of Clinical Sciences Lund, Faculty of Medicine, Lund University, 221 00 OrthopedicsLund, Sweden; 6 The Swedish Knee Arthroplasty Register, Lund, Sweden

**Keywords:** Obesity, Bariatric surgery, Osteoarthritis, Total knee arthroplasty, Patient-reported outcome

## Abstract

**Background:**

The impact of obesity on patient-reported outcome (PRO) after total knee arthroplasty (TKA) surgery has demonstrated varying results. We evaluated knee pain, Activity in Daily Life function (ADL), and satisfaction after TKA surgery in patients with and without prior bariatric surgery (BS).

**Methods:**

Scandinavian Obesity Surgery Registry (SOReg) and the Swedish Knee Arthroplasty Register (SKAR) were used to identify patients operated on with primary TKA for osteoarthritis (OA) between 2009 and 2019 that had a BS within 2 years before the TKA (BS group). These patients were compared to patients with TKA without prior BS (no BS group). The patients filled in the Knee injury and Osteoarthritis Outcome Score (KOOS) preoperatively and one year postoperatively as well as satisfaction with the surgery one year postoperatively. Multiple linear regression analysis was used to evaluate 1-year postoperative KOOS pain and ADL function between the 2 groups. Adjustments were made for sex, age, and preoperative KOOS pain and ADL function respectively.

**Results:**

Forty-four patients were included in the BS group and 3,525 patients in the no BS group. We found no statistically or clinically significant difference in one-year postoperative KOOS pain and ADL function between the BS group and the no BS group. The majority of the patients in both groups were classified as satisfied or very satisfied one year postoperatively to the TKA.

**Conclusions:**

Our results indicate that patients without BS prior to the TKA gain similar 1-year outcome in pain, ADL function and satisfaction as patients with prior BS.

**Graphical abstract:**

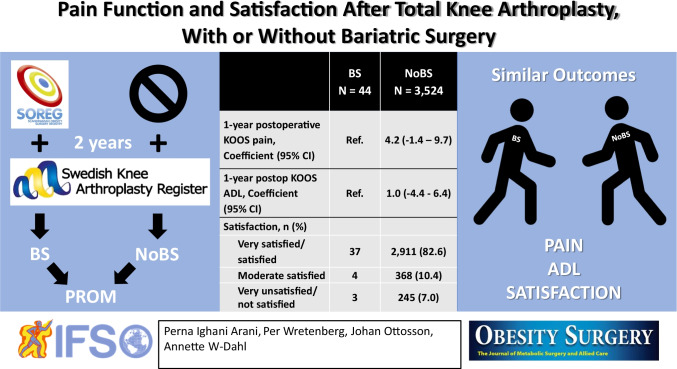

## Introduction

Obesity in the society is a medical challenge that continues to increase among the population in Europe [1]. Previous studies have shown that obesity has a negative impact on complications and mortality after total knee arthroplasty (TKA) [2–4]. Studies evaluating the impact of obesity on patient-reported outcome (PRO), such as pain and knee function, have demonstrated varying results [5, 6]. Our previous findings did not indicate that having a BS prior to TKA was associated with lower risk of revision [7]. Over time, the proportion of patients with obesity undergoing TKA has steadily been increasing [8]. We have recently published a study demonstrating that patients with BS prior to TKA had an increased risk of revision due to infection, compared to patients without prior BS. The control group consisted of patients within the same BMI range before their TKA [7]. Consequently, it is of clinical interest to determine if these patients were less satisfied regarding pain and ADL function one year after TKA.

Bariatric surgery has been demonstrated to be an effective method of reaching significant long-term weight loss in comparison with nonsurgical interventions in patients with obesity [9]. There are no previous studies examining the association of BS on PROs postoperatively to TKA. Therefore, the aim of this study was to evaluate pain and ADL function and satisfaction one year postoperatively in patients having BS before the TKA and compare them to TKA patients without BS.

## Patients and Methods

We used a subset of a cohort including patients who underwent primary gastric bypass or sleeve gastrectomy (BS) in 2007–2019 identified from the Scandinavian Obesity Surgery Register (SOReg) and patients having primary TKA for OA in 2009–2019 within 2 years after their BS, and patients without prior BS from the Swedish Knee Arthroplasty Register (SKAR). As previously described, we used the BMI prior to the TKA in the BS group, and selected the no BS patients within the same BMI interval (range 16.9–50) and age interval (41.9–67 years) as the BS group [7]. The SOReg was established in 2007 and the SKAR in 1975. The two registers have high completeness and correctness, validated through the national patient register [10, 11]. The subset consisted of patients having BS surgery and TKA surgery in the Region of Skåne, in the southern part of Sweden. The PROs (preoperatively and one year postoperatively) were obtained from the SKAR PROM project. The SKAR PROM project is voluntary and started in 2008 with one hospital and has extended to include 39 hospitals in 2020. Each of the 21 regions in Sweden has responsibility for the health care provided to the inhabitants. We included only the Region of Skåne as all hospitals in the region report PRO measures to the SKAR and BS is performed in the region as well. BMI, age, sex, and ASA classification were obtained from the SKAR.

**Fig. 1:**
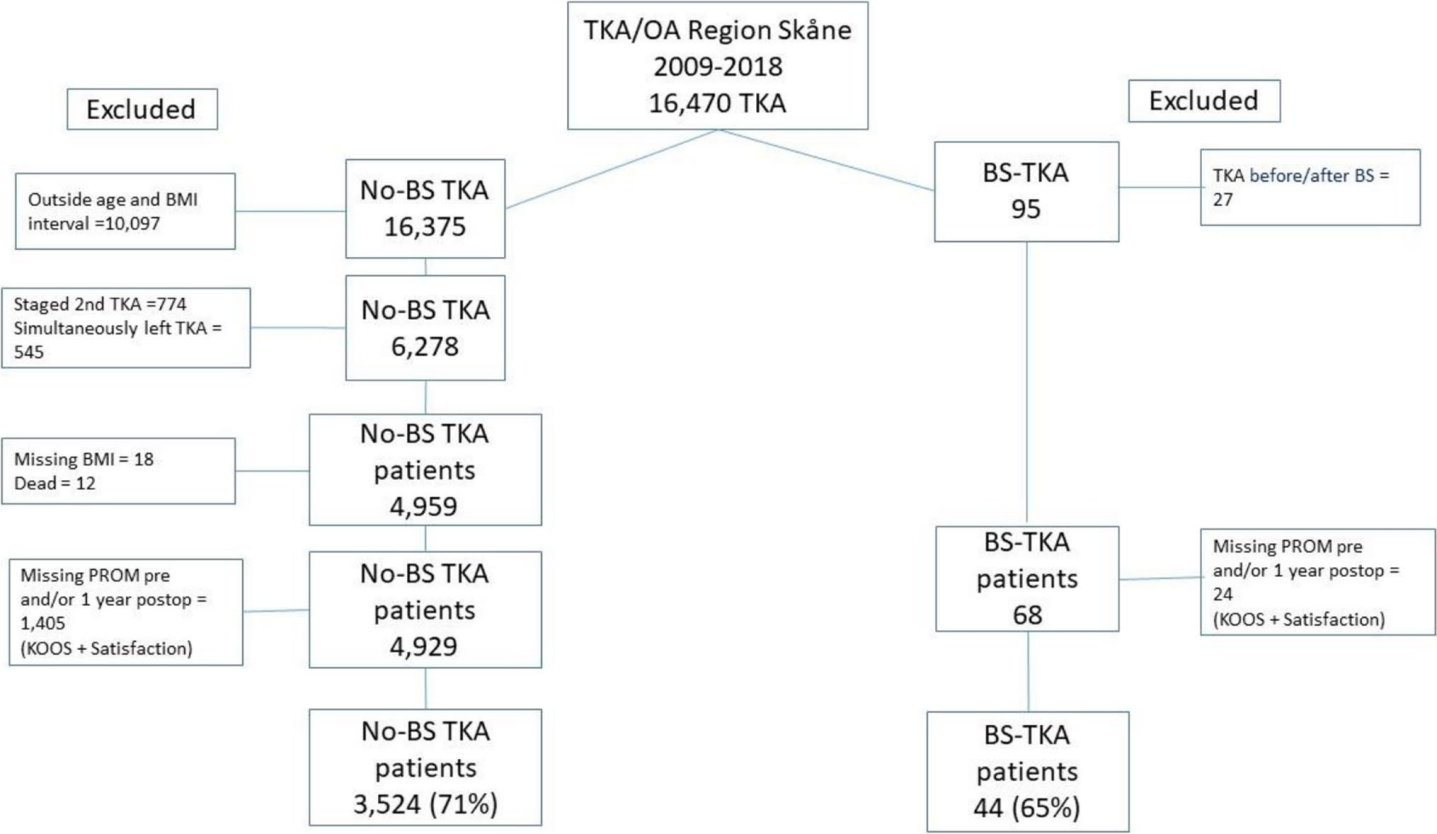
Flow-chart of the study population

In patients who had BS before the TKA (BS group), we excluded the 2^nd^ knee if both knees had been operated after the BS. In patients without prior BS to TKA (no BS group), we excluded the patients that were found in the SOReg. The 2^nd^ TKA were excluded in those who underwent staged bilateral surgery, and in patients who underwent simultaneous bilateral TKA, the left knee was excluded (Fig. 1). Only patients with complete PROs were included in the study (Fig. 1).

### KOOS

The Knee injury and Osteoarthritis Outcome Score (KOOS) is a patient-reported outcome measure (PROM), which assess the patient’s own experience about their knee and associated issues. It consists of 42 questions divided in 5 subscales: Pain, Symptoms, Activity in Daily Life function (ADL), Sport and Recreation Function (Sport/rec), and Quality of Life (QoL). Scores are transformed to a 0–100 scale, with 0 indicating extreme knee problems and 100 indicating no knee problems [12]. A score of ≥ 8 points in the KOOS was considered a clinically relevant difference for statistically significant results [13].

#### OMERACT-OARSI Responder

The Outcome Measures in Rheumatology (OMERACT) committee and The Osteoarthritis Research Society International (OARSI) Standing Committee for Clinical Trials Response Criteria Initiative have developed OMERACT-OARSI criteria, which are outcome measures for osteoarthritis that are based on the combination of absolute and relative changes in Western Ontario and McMaster Universities Osteoarthritis Index (WOMAC) pain, functional impairment, and total score one year postoperatively [14].

The KOOS was converted to WOMAC before classifying each patient by the OMERACT-OARSI criteria into high, low, or no responders one year postoperatively [10]. High responder is defined as a patient with an improvement of ≥ 20 points, which should also correspond to ≥ 50% improvement in pain or function, compared to preoperative WOMAC score. Low responder is defined as improvement of ≥ 10 points also corresponding to ≥ 20% in two of the WOMAC pain, function, or total score.

#### Patient Satisfaction

The patients’ satisfaction with the TKA surgery one year postoperatively was assessed by using a 0–100 Visual Analogue Scale, where 0 is the high satisfaction and 100 is no satisfaction. Depending on the score given, the patients were classified into 5 groups; very satisfied (0–20), satisfied (21–40), moderately satisfied (41–60), not satisfied (61–80), and very unsatisfied (81–100).

#### Statistics

Patient characteristics were presented as means with standard deviations (SD) or numbers and percent when appropriate. The KOOS subscales were presented as means with 95% confidence intervals (CI).

Multiple linear regression analysis was performed to evaluate one-year postoperative pain and ADL function between the 2 groups. We adjusted for sex, age and BS or not, and preoperative KOOS pain and ADL function respectively. BMI was not included as a confounder since the difference between the cohorts was only one unit.

Statistical analyses were carried out using Stata version 15 (StataCorp, College Station, TX, USA).

#### Ethics, Funding, and Potential Conflicts of Interest

The study was conducted according to the declaration of Helsinki [15], and was also waived by the Swedish Ethical Review authority (2017/466). Informed consent does not apply. The authors have no conflicts of interest to report.

## Results

Forty-four patients operated on with bariatric surgery prior to the TKA for OA were included in the BS group and 3,524 patients without BS were included in the no BS group. The BS group was younger (mean age: 56 vs 60 years) and had a higher proportion of females (86% vs 57%) compared to the no BS group. The majority of patients had an ASA score of 2 in BS and no BS group (70% and 62% respectively) (Table 1). The BS group had a mean reduction of 14.2 (SD 3.3) in BMI after BS, and a mean total weight loss of 33.6% (SD 12%). The mean time between BS and TKA was 1.1 years (SD 0.43).

**Table 1:** Patient characteristics and preoperative KOOS for included and excluded/missing TKA patients with (BS) or without prior BS (no BS)

	BS *N* = 44	No BS *N* = 3,524	*p* values
Age in years, mean (SD)	55.9 (5.7)	60.4 (5)	< 0.001
Sex, *n* (%)			< 0.001
Female	38	2,024 (57.4)	
Male	6	1,500 (42.6)	
ASA classification,* n* (%)			< 0.001
1	7	1,028 (29.2)	
2	31	2,175 (61.8)	
≥ 3	6	318 (9.0)	
BMI, mean (SD)	30.6 (3.7)	29.5 (4.4)	0.053
Preoperative KOOS score, mean (CI)			
Pain	32 (29–36)	37 (37–38)	0.012
ADL	43 (39–47)	45 (44–46)	0.409
Symptom	37 (32–42)	42 (41–43)	0.050
Sport/rec	6 (3–9)	10 (9–10)	0.008
QOL	19 (16–23)	20 (20–20)	0.767

*SD*, standard deviation; *CI*, 95% confidence interval; *ASA*, American Society of Anaesthesiologists

The BS group reported statistically significant worse KOOS scores 1-year postoperatively for pain, but not for ADL (Pain: 71 (CI 64–77), ADL: 75 (CI 69–82)) than the no BS group (Pain: 78 (CI 77–79), ADL: 78 (CI 78–79)), but without clinically relevant differences (Table 2). When adjusting for sex, age and BS or not, and preoperative KOOS pain and ADL function, respectively, we found no statistically significant difference between the groups (Pain: 4.2 (CI 1.4–9.7), ADL: 1.0 (CI 4.4–6.4)) (Table 3). The majority of the BS and the no BS group were high responder according to the OMERACT-OARSI criteria and were classified into satisfied or very satisfied one year postoperatively to TKA (Table 2).

**Table 2:** One-year postoperative KOOS, OMERACT-OARSI responders, and satisfaction with the surgery in TKA patients with prior BS or without prior BS

	BS *N* = 44	No BS *N* = 3,524	*p* value
Postoperative KOOS score, mean (CI)			
Pain	71 (64–77)	78 (77–79)	0.033
ADL	75 (69–82)	78 (78–79)	0.337
Symptom	68 (62–73)	72 (72–73)	0.100
Sport/rec	32 (24–40)	35 (34–36)	0.454
QOL	59 (52–66)	60 (60–61)	0.623
OMERACT-OARSI responder,* n* (%)			
High responder	36	2,820 (80,0)	
Low responder	3	293 (8,3)	
No responder	5	411 (11,7)	
Satisfaction,* n* (%)			
Very satisfied/satisfied	37	2,911 (82.6)	
Moderate satisfied	4	368 (10.4)	
Very unsatisfied/not satisfied	3	245 (7.0)	

*CI* 95% confidence interval

**Table 3:** The relationship between BS or no BS prior to TKA and one-year postoperative KOOS pain and ADL function respectively

1-year postoperative KOOSpain*	1-year postoperative KOOS ADL*
Coefficient (95% CI)	Coefficient (95% CI)
BS (*N* = 44) reference	Reference
No BS (*N* = 2,524) 4.2 (− 1.4 to 9.7) *p* = 0.145	1.0 (− 4.4 to 6.4) *p* = 0.717

^*^Adjusted for age, sex, and preoperative KOOS pain and ADL function respectively

## Discussion

We found no clinically relevant difference in one-year postoperative pain and ADL-function nor satisfaction and OMERACT-OARSI responder between patients with BS prior to the TKA and those without BS. To our knowledge, this is the first study investigating the association between BS or no BS prior to TKA and PROs.

However, our study has limitations. We included relatively few patients in the BS group indicated by the relatively wide CIs, which mean that we may not be able to show differences that may exist. The rationale to include only one region was that the SKAR PROM project has PRO measures from the Region of Skåne since 2008, and BS is also performed in the region. Furthermore, it is an observational study with limited variables included. With the relatively low number of TKA patients with prior BS, we limited our analysis and used the available variables relevant as potential confounders.

However, the data from the SOReg and the SKAR is prospectively collected and both registers are known for having a high completeness and quality [10, 16].

The mean age of patients undergoing BS in Sweden is slightly above 40 years, and a majority of these patients are women [16]. This is in line with the cohort included in the current study. In order to make the groups more comparable, we selected the patients in the no BS group within the same age interval as those in the BS group. When filtering the selection of the no BS group regarding BMI, we used the BS groups BMI prior to the TKA. We did not include BMI in the regression analysis as the difference in BMI was only one unit. We decided not to adjust for the ASA classification as BMI interferes with comorbidity [17], and BMI is in addition a factor in deciding the ASA classification. A majority of the patients in both cohorts were classified to ASA 2, which demonstrate that most of the patients were relatively healthy or suffered from a mild systemic disease. We did a sensitivity analysis were we included ASA as confounder; however it did not change the results. The cohort includes the two most common bariatric surgeries in the BS group; gastric bypass and sleeve gastrectomy (95% and 5% respectively). Due to low number of patients in the BS group, no subgroup analysis was done.

A majority of the patients in both groups were high responder and “very satisfied” or “satisfied” one year postoperatively to TKA. Overgaard et al. (2019) compared PROs in different BMI categories and showed similar satisfaction rates among the BMI categories. Compared to mean values, which can be affected by outliers and hide both good and bad outcome, the OMERACT OARSI responder presents a clearer picture of the patients’ improvement from given criteria.

Obesity has been associated to worse outcomes in patients undergoing TKA, primarily when it comes to the risk of revision [18]. During the past decades, the proportion of patients with obesity undergoing primary TKA has increased in the USA, resulting in a higher incidence of revision surgeries. However, this has not been the case in Sweden where the proportion of patients with *BMI* ≥ 35 (9.3%, 2019) having a knee arthroplasty has slightly decreased during the last decade [10, 19]. An effective method of achieving long term weight loss is bariatric surgery [9]. Consequently, BS may reduce the risk of revision for patients having a subsequent TKA. However, in a previously study, we found the risk of revision for any reasons and for infection not to be reduced in patients having a TKA due to osteoarthritis within 2 years after BS compared to those without BS [7].

Studies on PROs after TKA in patients with obesity have shown varying results [5, 6]. Overgaard et al. (2019) evaluated the KOOS one year postoperatively to TKA and found no clinically relevant difference in pain or ADL function between different BMI groups in over 3,000 patients. On the other hand, Xu et al. (2018) showed that the patients with obesity had statistically significant poorer Oxford Knee Scores 10 years after TKA surgery when compared to those without obesity in 126 patients. However, the patients being 10 years older in the follow up may influence the outcome.

It has been postulated that weight loss improves the outcomes after TKA; both dietary and surgical methods have been proposed and tested [20–22]. In a randomized pilot trial on 40 patients with BMI ≥ 30 who were undergoing a total hip and knee replacement, the effects of a dietetic intervention were compared to usual care. An association between maintained or lost weight and better outcomes in pain and activity was demonstrated compared to patients who had gained weight one year after the intervention. However, when comparing the intervention group to the control group, they found no difference in pain, function, or activity levels [20]. Keeney et al. (2019) assessed physical function improvement in 203 morbidly obese patients, where the patients were divided into groups depending on the weight reduction (5, 10, and 20 pounds of reduction in weight) and found no differences in physical function outcome between the groups [21]. Furthermore, Liljenso et al. (2019) investigated the effect of 8-week low energy diet on 76 TKA patients in a randomized control trial. Despite the fact that patients in the diet group maintained their weight loss one year after surgery, they found no statistically significant difference in either quality of life or knee function. However, the mean reduction was only 3.2 BMI units in the intervention group [22]. The weight reduction in these studies is much lower in the intervention groups compared to the expected weight loss after BS.

A systemic review on the effects of BS on knee complaints in patients with morbid obesity found that patients with morbid obesity undergoing BS with subsequent marked weight loss are likely to improve knee pain, physical function and stiffness. [23]. In addition, Parvizi et al. (2000) found that the Knee Society Score improved significantly 2–6 years after TKA in 7 patients with prior BS. [24]. The abovementioned studies included relatively few patients which may influence the possibility to find differences that may exist.

Some of the results mentioned are in line with our results showing no relevant difference in knee pain or function between the groups receiving intervention for weight loss and those without interventions. However, none of the discussed studies had the same objective, nor patient groups as our study, and they included as well relatively few patients. The mentioned studies also differ from our study in their objectives. In this current study, we have analyzed how the BS in itself affects PROM, and not the weight loss, in comparison to the mentioned studies.

## Conclusion

Our study may indicate that patients having BS prior to their TKA have similar one year outcome regarding their pain and ADL function as compared to patients within the same age and BMI interval without BS. However, our BS group consisted of relatively few patients and future studies with larger sample size are needed.
